# ZIF-8 and Its Magnetic Functionalization as Vehicle for the Transport and Release of Ciprofloxacin

**DOI:** 10.3390/pharmaceutics14112546

**Published:** 2022-11-21

**Authors:** Ventura Castillo Ramos, Cinthia Berenice García Reyes, Guillermo Mangas García, María Inmaculada Sampedro Quesada, Fernando José Martínez-Checa Barrero, Jacob Josafat Salazar Rábago, Manuel Sánchez Polo

**Affiliations:** 1 Department of Inorganic Chemistry, Faculty of Science, University of Granada, 18071 Granada, Spain; 2 Faculty of Chemical Sciences, Nuevo León Autonomous University, San Nicolás de los Garza 66455, Mexico; 3 Department of Microbiology, Faculty of Pharmacy, University of Granada, 18071 Granada, Spain

**Keywords:** metal organic frameworks, magnetic ZIF-8, drug release, ciprofloxacin, pharmaceutical

## Abstract

The use of nanomaterials for the controlled release of drugs aims to enhance their effectiveness, especially when poorly soluble in water, and achieve their rapid, localized, and effective administration. The present study focuses on the use of a Zeolitic Imidazolate Framework-8 (ZIF-8) as vehicle for the transport and controlled release of the antibiotic ciprofloxacin (CIP) as model due to its favorable physicochemical characteristics. The objective is to synthesize the ZIF-8 material loaded with CIP through encapsulation for subsequent release of the drug in neutral and acid physiological media. In addition, functionalization of the CIP/ZIF compound with magnetic nanoparticles (NP) was sought to increase its traceability through the possible use of magnetic fields. Characterizations by XRD, FT-IR, SEM-EDX, and TGA showed a satisfactory synthesis of both pure ZIF-8 and ciprofloxacin-loaded ZIF-8, with high crystallinity and thermal stability. The release profiles showed an abrupt initial release that stabilized over time. A much higher release (20–80% greater) was obtained in acid versus neutral pH in all cases, attributable to the collapse of the ZIF-8 structure in acid media. In addition, functionalization of the material with iron NPs did not affect the behavior of the system during drug release. Antimicrobial activity tests against *E. coli* and *S. aureus* showed that ZIF-8 per se exerts antimicrobial activity, while the compounds CIP/ZIF and magnetic CIP/ZIF increased the antimicrobial capacity of pure CIP by 10–20%. The ZIF-8 system has high potential as a drug carrier and release agent for the treatment of diseases, especially those that cause acidification of the cellular environment, achieving a rapid, localized, and targeted action with the possibility of achieving traceability of the system after its magnetic functionalization.

## 1. Introduction

**Figure 1:**
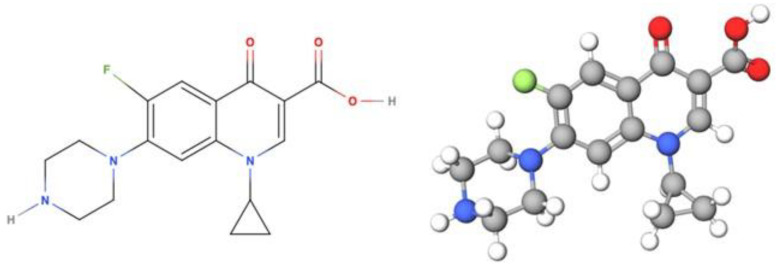
Structure of ciprofloxacin.

Ciprofloxacin (CIP) is an antibiotic in the group of second generation quinolones, also known as quinoline derivatives (Figure 1). It exerts bactericide activity by inhibiting topoisomerases II and IV and presents a broad spectrum of action against Gram-positive and Gram-negative bacteria and mycobacteria. For these reasons, it is one of the most widely used fluoroquinolones and is described by the World Health Organization as an essential drug for a basic health system [1,2,3].

Its most frequent applications include urinary infections and pneumonia, being administered orally at different doses when possible and intravenously in more severe cases [4,5,6]. As in other antibiotics, one of the main challenges is the possible generation of resistances that reduce the availability of the drug or prevent its effective action [7,8,9]. CIP has a water solubility of 1.35 mg/mL at 25 °C [10], i.e., slightly soluble according to the United States Pharmacopeia and National Formulary [11].

Various technologies have been developed to administer drugs with low water solubility, including the use of excipients to improve solubility (surfactants, complexing agents, lipid formulations, etc.) [12]; solid dispersion and drug crystal size reduction [13]; and, more recently, the use of nanomaterials as carriers for the controlled administration and release of drugs over time, which also offers a solution to the problems of availability and resistance, reducing adverse effects [14]. Targeted drug administration with nanomaterials is of major importance for diseases in which the required dose cannot be administered in the usual manner due to low blood circulation in hard tissues, among other reasons [15].

The use of drug-loaded nanomaterials for biomedical applications is of particular interest. Their advantages include the following: nanometric size; the possibility of synthesis with different morphologies to mimic the biological environment in which they are administered; the ability to control their physicochemical properties and modify/functionalize their surface in a simple manner [16,17]; and even the capacity to reduce the toxicity of certain drugs, thereby optimizing the dose required and minimizing their possible bioaccumulation in water media [18].

Metal Organic Frameworks (MOFs) are crystalline solids with a porous structure formed by a network of metal centers connected by organic ligands. The large surface area, adjustable pore size, and ready functionalization of MOFs affords them the potential to carry a wide range of large molecular size drugs for their subsequent controlled release [19,20,21].

**Figure 2:**
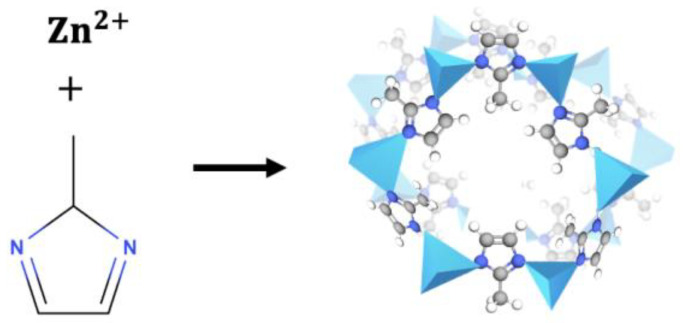
Schematic representation of the structure of ZIF-8.

Numerous MOFs are available with different crystalline structures and physicochemical properties as a function of the metal centers and organic ligands employed for their synthesis. One MOF, Zeolitic Imidazolate Framework-8 (ZIF-8), comprises Zn^2+^ metal centers connected by nitrogen atoms from an organic imidazole molecule (organic ligand), forming organometallic structures (Figure 2) [22]. Its favorable physicochemical properties (pore size and volume) allow it to carry high molecular weight molecules such as drugs or therapeutic agents. It is also pH-sensitive, degrading at slightly acid pH values (around 4.5–5) and remaining more stable in neutral physiological media [23]. They can, therefore, be of value to release drugs in diseases that course with cell acidification, enabling the localized and specific release of the therapeutic agent. Based on these characteristics, ZIF-8 was selected as the CIP carrier material for the present study.

The study objectives were as follows: to carry out ZIF-8 synthesis and simultaneous CIP encapsulation in a single step for utilization in drug release; to evaluate the antibacterial capacity of the materials; and to explore functionalization of the CIP/ZIF-8 system with magnetic particles to enhance its applicability.

## 2. Materials and Methods

### 2.1. Materials Characterizations

X-ray diffraction (XRD) was performed in a Bruker D8 DISCOVER diffractometer with DECTRIS PILATUS3R 100K Detector (Madrid, Spain). Samples were placed between glass panels and compressed into thin layers to avoid noise in the XRD pattern. A step size of 2θ 0.02° was used in the irradiation to obtain diffractograms of 5° < 2θ < 50°. The simulated crystal information for the magnetite (Fe_3_O_4_) was provided by the Crystallography Open Database (COD) with its corresponding COD number and was plotted by VESTA software together with the experimental XRD patterns. The infrared spectra were determined in a Fourier-Transform Infrared Spectrometer, JASCO 6200 (Tokyo, Japan), with SPECTRA MANAGER v2 software, using the Attenuated Total Reflectance (ATR) accessory. A small amount of sample was placed on the measurer and compressed using a diamond-tipped endless screw. An average of 64 scans were gathered for each spectrum from 420 to 4000 cm^−1^ at a nominal resolution of 1 cm^−1^. Thermogravimetric analyses (TGAs) were obtained in a TGA-50H SHIMADZU (Greifensee, Switzerland) thermogravimetric analyzer with vertical design and maximum precision of 0.001 mg. The samples were ground and dried in the furnace at 60 °C overnight before the analysis. TGA diagrams were obtained in air atmosphere, with a heating ramp of 5 °C/min up to 950 °C. SEM images were captured in a HITACHI S-510 Scanning Electron Microscope (Austin, TX, USA) with a Röntec, M Series Edwin, Si EDX detector, (Li). A small amount of powdered material was dropped onto a carbon tape on the SEM support, and the sample adhering to the carbon tape was then dried in the furnace and coated in gold.

### 2.2. ZIF-8 Synthesis

ZIF-8 was synthesized with hexahydrate zinc nitrate Zn(NO_3_)_2_·6H_2_O (SIGMA-ALDRICH, reagent grade, 98%, St. Louis, MO, USA) as Zn metal center precursor and 2-methylimidazole (2-MeIm, SIGMA-ALDRICH, 99%, St. Louis, MO, USA) as organic ligand precursor in a molar proportion of 1:8, using distilled deionized (DDI) water or acetone (ACS BASIC) as solvents to study their effect on MOF formation [24].

Both zinc nitrate and 2-methylimidazole were dissolved in the solvent (DDI water or acetone) separately under agitation, adding 2-meIm dimethyl sulfoxide (Panreac, PRS, Barcelona, Spain) to the solution to favor its dissolution in water. Next, while maintaining agitation, the zinc solution was rapidly added to the 2-MeIm solution at room temperature. The reaction was maintained for 5 min or 24 h to examine the effect of reaction time on MOF formation. The removed material was then centrifuged (Hettich Universal 320 R, Darmstadt, Germany) for 15 min at 14,000 rpm and washed three times with ethanol (96% Analytical Grade ACS, Darmstadt, Germany) and methanol (HPLC grade ACS, Darmstadt, Germany), followed by overnight drying at 65 °C in a conventional oven (ARGO LAB TCF 120, Carpi MO, Italy). The same procedure was followed for ZIF-8 synthesized in DDI water or acetone. Accordingly, four different materials were prepared: ZIF-8 (5 min, synthesis in DDI water); ZIF-8 (24 h, synthesis in DDI water); ZIF-8 (5 min, synthesis in acetone); and ZIF-8 (24 h, synthesis in acetone).

### 2.3. CIP Encapsulation in ZIF-8

**Figure 3:**
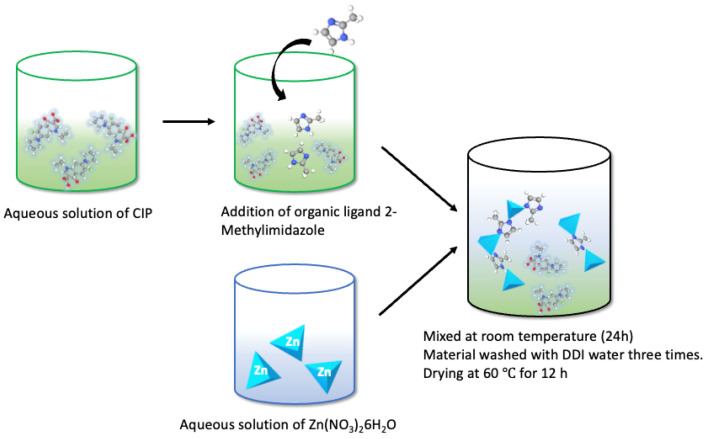
Outline of the in situ encapsulation of CIP in ZIF-8.

CIP was encapsulated (Sigma, ≥98%, HPLC, St. Louis, MO, USA) within ZIF-8 by its addition during MOF synthesis, using the ZIF-8 with reaction time of 24 h synthesized in DDI water. First hexahydrate zinc nitrate and then CIP (10 mg, 100 mg, or 1000 mg) were dissolved in DDI water. The 2-MeIm solution was added to the CIP solution (see proportions above), and, while maintaining agitation, the zinc solution was added to the CIP solution along with organic ligand 2-MeIm. After agitating for 24 h, ZIF-8 was formed with the drug encapsulated within its structure, having a milky white color (Figure 3). The compound is designated CIP/ZIF-8 (drug load, mg/mg).

### 2.4. Iron Nanoparticle Synthesis

Briefly, 3.25 g hexahydrate FeCl3 (PROBUS S.A., 98–102%, Zeppelinstraße, Germany) and 1.67 g heptahydrate FeSO_4_ (SIGMA-ALDRICH, ReagentPlus ≥ 99%, St. Louis, MO, USA) were completely dissolved in 50 mL DDI water at 50 °C under strong agitation for 30 min. The temperature was then increased to 75 °C, adding 6.25 mL 25% ammonium solution (as NH_3_, pure pharma grade) under agitation for a further 60 min. Next, 6.25 mL 1.5 M trisodium citrate (MERCK, Pharmaceutical Secondary Standard, St. Louis, MO, USA) was added under agitation at 85 °C for 1.5 h in N_2_ atmosphere to avoid nanoparticle oxidation. The resulting black precipitate was separated from the solution and removed by immersing a magnet for 5 min, and it was then washed with water and ethanol and dried overnight at 60 °C, obtaining a solid, dark (almost black) product [25].

### 2.5. Magnetic ZIF-8 Synthesis

For the synthesis of magnetic ZIF-8, iron nanoparticles were added during ZIF-8 synthesis at the same time as CIP encapsulation, thereby synthesizing magnetic ZIF-8 with encapsulated CIP in a single step; 0.112 g zinc nitrate was dissolved in 1 mL DDI water and 1.026 g 2-MeIm with 100 mg CIP in 10 mL DDI water.

In addition, 20 mg of iron nanoparticles were dispersed in 2 mL DDI water for 15 min under agitation until homogeneously dispersed. Subsequently, the magnetic nanoparticle solution was mixed with the zinc and 2-MeIm solutions, agitating for 30 min at room temperature. The material was then centrifuged at 6000 rpm for 10 min, and the precipitate was washed three times with DDI water and set to dry all night at 60 °C, obtaining a solid dark brown product.

Accordingly, two materials were synthesized, magnetic ZIF/CIP and magnetic ZIF-8, with and without drug, respectively, adding the CIP or not during the aforementioned synthesis stage.

### 2.6. CIP Release in Acid and Neutral Media

Two media were prepared for drug release: a pH 7.4 phosphate buffer, representing healthy human cells; and a pH 5 acetate buffer, representing the slightly acidified cells in some diseases. Release tests of the ZIF-8-encapsulated drug used 20 mg of each material (low, medium, and high dose) in duplicate in 11 mL of the acid or neutral buffer, maintaining a temperature of 37 °C in a double boiler on a heating plate. Aliquots of 0.5 mL were withdrawn at different time points (from 5 min to several days), refilling the original solution with the same amount of corresponding buffer. Withdrawn volumes were centrifuged at 10,000 rpm for 5 min to halt the release. UV spectra were subsequently measured (VWR UV-1600PC Spectrophotometer, Santa Clara, CA, USA) at each pH, detecting the CIP peak (271 nm in phosphate buffer and 277 nm in acetate buffer) at the different time points and transforming the data by using previously prepared standard curves.

### 2.7. Release Kinetics

The release profiles obtained were adjusted to zero-order, first-order, Higuchi, and Korsmeyer–Peppas kinetic models [26], which are described in detail in the Supplementary Information.

### 2.8. Antimicrobial Activity Test

The microorganisms used in this study were the following: *Staphylococcus aureus* (ATCC 9144) and *Escherichia coli* (CECT 101). A suspension was prepared from 24 h culturing of each bacterium in normal saline, containing 1.5 × 10^8^ CFU/mL bacteria in accordance with the 0.5 McFarland standard. The test was carried out through the standard diffusion disk method based on the CLSI (2014) protocol [27]. The bacterial suspensions were spread on Petri dishes prepared using Mueller–Hinton agar (Merck, Germany). Cellulose discs (6 mm) containing 5 mg of pure ZIF-8 and ciprofloxacin-loaded ZIF-8 were disposed on the inoculated Petri dishes. Ciprofloxacin (5 mg) was used as positive control. The Petri dishes were incubated at 37 °C for 24 h. After this incubation time, the antibacterial effect was measured by comparison of the exclusion zone around the cellulose discs for the different materials respect to the positive control.

## 3. Results

### 3.1. Materials Characterizations

The synthesized materials were physiochemically characterized to determine their composition, size, morphology, crystallinity, and thermal stability.

**Figure 4:**
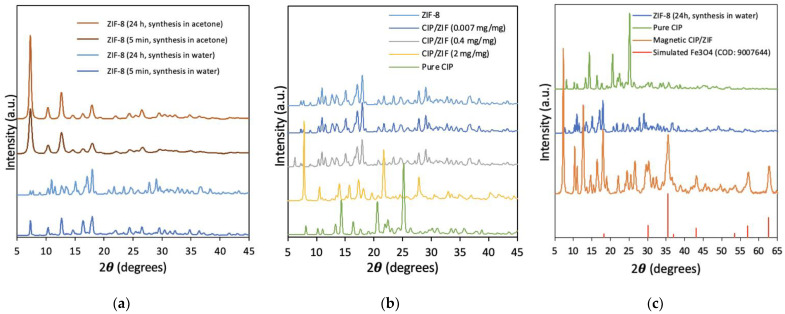
X-ray Diffraction patterns of (**a**) ZIF-8 with different synthesis conditions; (**b**) ZIF-8 and encapsulated CIP in ZIF-8 at different loadings; and (**c**) magnetic CIP/ZIF material.

XRD was used to determine the crystallinity of the materials and confirm their correct synthesis. As observed in Figure 4a, the use of different solvents and reaction times influenced the crystallinity. For ZIF-8 synthesized in acetone, the main characteristic diffraction peaks appeared at 2θ = 7.4°, 10.4°, 12.7°, and 18.0° corresponding to planes (011), (002), (112), and (222), respectively [28]. Greater crystallinity was observed in the sample synthesized in acetone for 24 h versus 5 min. For ZIF-8 synthesized in DDI water for 5 min, virtually the same diffraction peaks were observed, characteristic of spherical ZIF-8, although with lower crystallinity. However, new peaks appeared in the diffractogram for ZIF-8 synthesized in DDI water for 24 h in water, indicating a change in the crystalline morphology of ZIF-8 from a spherical to a “flower” shape, in agreement with previous reports [24]. This change was confirmed by the SEM images obtained.

Figure 4b depicts the XRD of ZIF-8 synthesized in water for 24 h with different loads of encapsulated CIP (high, medium, and low). The crystallinity of CIP/ZIF (0.007 mg/mg) and CIP/ZIF (0.4 mg/mg) materials (low and medium loads) was similar to that of pure ZIF-8, suggesting that the presence of the drug did not affect synthesis of the MOF, which could form its characteristic flower shape with no change in the crystallinity, housing the drug in its structure. In the case of CIP/ZIF (2 mg/mg) (high load), however, some diffraction peaks appeared and others disappeared in comparison to pure ZIF-8, indicating that the presence of an elevated drug concentration negatively affects the formation of the crystalline structure of ZIF-8. SEM images also revealed that CIP/ZIF (2 mg/mg) did not have its characteristic flower shape.

Figure 4c shows that magnetic CIP/ZIP (0.6 mg/mg) has the characteristic diffraction peaks of pure ZIF-8 and some peaks corresponding to CIP, alongside the high-intensity peak attributed to magnetite (Fe_3_O_4_) at 37 °C [25], as can be observed from its simulated pattern (COD: 9007644) (Figure S2). These findings suggest that no significant changes in the material structure result from the drug encapsulation and functionalization with iron nanoparticles.

**Figure 5:**
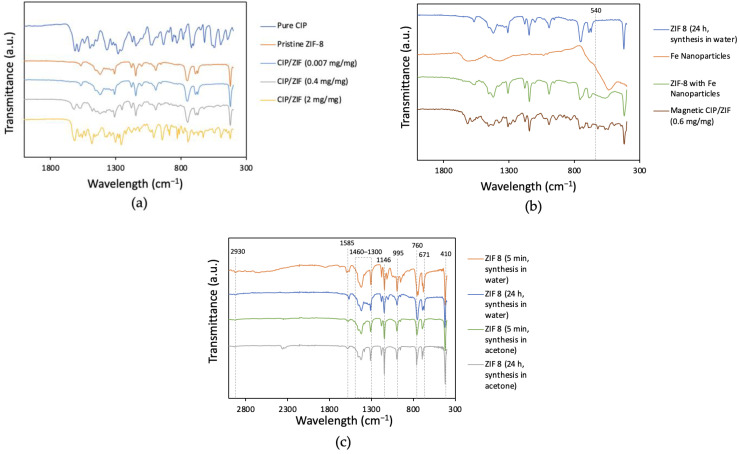
FT-IR spectra of (**a**) ZIF-8 and encapsulated CIP in ZIF-8 at different loadings; (**b**) magnetic CIP/ZIF material and Fe NP; and (**c**) ZIF-8 with different synthesis conditions.

Figure 5a,c depicts the characteristic infrared spectrum of ZIF-8: 2930 cm^−1^ (asymmetrical stretching vibrations of aliphatic groups C-H); 1585 cm^−1^ (C=N elongation vibration); the region between 1300 and 1460 cm^−1^ (aromatic rings); 1146 cm^−1^ (stretching of the aromatic group C-N); 995 cm^−1^ (folding of aromatic group C-N); 760 cm^−1^ (folding of aromatic group C-H); and 671 cm^−1^ (imidazole ring of the organic ligand). The peak at 410 cm^−1^ corresponds to the stretching of group Zn-N, showing the chemical composition of Zn bound to the nitrogen atom of the organic ligand [28]. The infrared spectra of CIP/ZIF (0.007 mg/mg) and CIP/ZIF (0.4 mg/mg) (low and medium loads) were similar to that of pure ZIF-8. However, the material with the highest drug load CIP/ZIF (2 mg/mg) obtained a combined spectrum between ZIF-8 and CIP, yielding peaks characteristic of both species. The finding of CIP on the surface of the material suggests that this high dose of the drug is not feasible for encapsulation within the structure of ZIF-8.

Figure 5b shows that the characteristic peak for the Fe-O group stretching at 540 cm^−1^ [25] appears in the ZIF-8 sample functionalized with Fe nanoparticles and in the sample corresponding to the encapsulated drug. This confirms the successful addition of Fe NP to the MOF surface. The sample of magnetic ZIF-8 with the encapsulated drug also shows the peaks characteristic of CIP, corroborating the formation of the magnetic ZIF-8 composite with CIP encapsulated within its structure.

Figure 5c shows that the characteristic chemical composition of ZIF-8 is independent of the solvent and reaction time, as previously observed by authors using different solvents for ZIF-8 synthesis [28].

**Figure 6:**
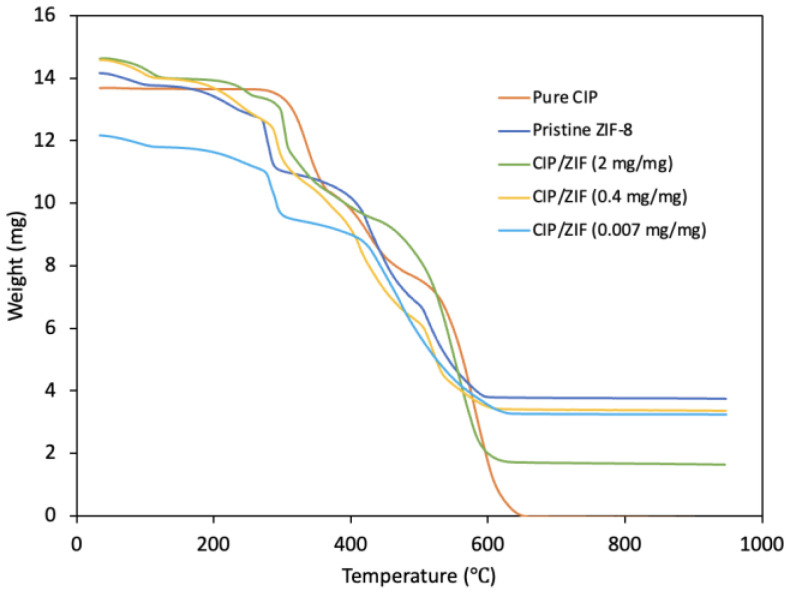
Thermogravimetric analysis of pure CIP, pure ZIF-8, and CIP/ZIF-8 with low, medium, and high drug loads.

The TGA results (Figure 6) show a first fall below 200 °C due to the removal of adsorbed water from the cavities of the structure and a second fall at around 300 °C in pure ZIF-8, corresponding to the removal of the organic ligand that has not reacted and a starting point of ZIF-8 decomposition. The most abrupt fall is at around 400–600 °C, indicating elimination of the organic part of the MOF and the collapse of its structure. Hence, ZIF-8 is stable up to 300 °C.

According to the curve for CIP, its thermal decomposition begins at around 300 °C. The materials with encapsulated CIP show a similar profile, produced by the drug decomposition, although this is less evident for the material with the lowest load, whose profile is more similar to that of pure ZIF-8. The first falls in the decomposition curves of materials with encapsulated CIP are attributable to removal of the water contained in its structure and used as solvent in the synthesis. It can be confirmed that encapsulated CIP/ZIF materials are stable up to around 300 °C.

**Figure 7:**
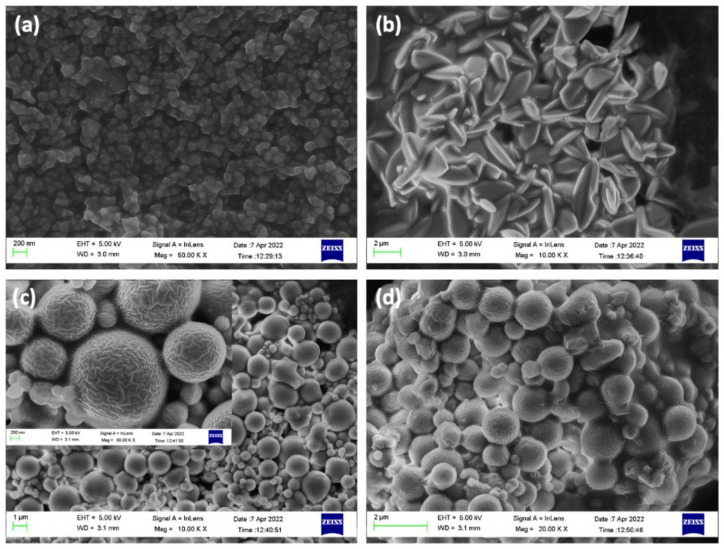
SEM images of (**a**) ZIF-8 (5 min, synthesis in water); (**b**) ZIF-8 (24 h, synthesis in water); (**c**) ZIF-8 (5 min, synthesis in acetone); and (**d**) ZIF-8 (24 h, synthesis in acetone).

**Figure 8:**
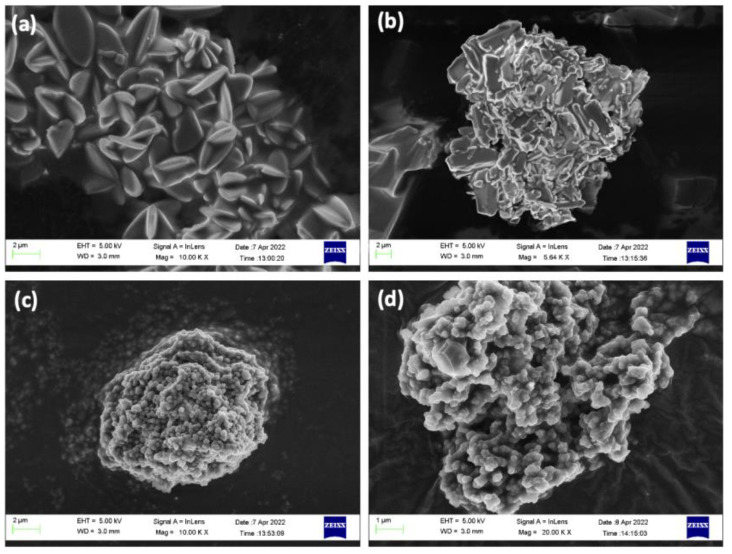
SEM images of (**a**) CIP/ZIF (0.007 mg/mg); (**b**) CIP/ZIF (2 mg/mg); (**c**) magnetic ZIF-8; and (**d**) magnetic CIP/ZIF (0.6 mg/mg).

High-resolution SEM images (Figure 7 and Figure 8) display the differences among ZIF-8 synthesis processes, highlighting the compound used for the encapsulation and adsorption of ZIF-8 synthesized for 24 h in DDI water, which is 2 µm and “flower-shaped” due to the use of water as a solvent, while the synthesis in water for 5 min is 0.1 µm in size and close to spherical (but not well defined). However, the synthesis with acetone forms well defined spherical structures with a very wide range of sizes, 0.1 to 1.5 µm, with similar distribution in both 5 min and 24 h, although it has the disadvantage of forming aggregates.

SEM images of compounds with low loads of encapsulated CIP display their typical flower structure, indicating satisfactory drug encapsulation (Figure 8a). It can be seen that ZIF-8 cannot satisfactorily encapsulate the drug and form its crystalline structure when the dose is increased to 2 mg/mg (Figure 8b). This is due to the high concentration of the drug in the solution and the formation of aggregates that increase its size, preventing its encapsulation within the structure of the MOF incapable of encapsulating it inside its structure. Figure 8c,d show that Fe nanoparticles inhibit aggregation showing similar structures of 0.1–0.2 µm with almost spherical features. Therefore, the coating of iron nanoparticles gives magnetic ZIF-8 a more spherical morphology, both without (Figure 8c) and with CIP encapsulated in its structure (Figure 8d). Figure 4c XRD spectrum for “Magnetic CIP/ZIF” also agree well with the “ZIF-8 24 h synthesis” peaks with the additional Fe_3_O_4_ peaks from its simulated pattern.

Energy-dispersive X-ray (EDX) analysis was used to determine the elements on the surface of the materials (Figure S1), which all showed the elements of ZIF-8 including zinc, and carbon and oxygen from the organic ligand 2-MeIm. We highlight the finding of the peak with fluoride, corresponding to CIP, for the material with the highest antibiotic load, indicating the presence on the surface of CIP crystals that had not been correctly encapsulated. Peaks corresponding to iron are evident in the spectra for magnetic ZIF-8.

The size, morphology, and crystallinity of ZIF-8 are influenced by the solvent selection and reaction time [24], which can be adjusted to scale the material to nanometric size and create a morphology suited to the biological environment in which it acts. The incorporation of Fe_3_O_4_ nanoparticles influenced the size, morphology, and crystallinity more drastically showing that the 24 h synthesis with nanoparticles produced features similar to the 5 min process without nanoparticles. In the present study, the material used for all encapsulation/adsorption experiments was ZIF-8 synthesized in DDI water for 24 h because it offered greater flexibility for the drug encapsulation and tests. However, this does not rule out the optimization of any of the aforementioned materials for the same purpose.

### 3.2. Release Profiles

**Figure 9:**
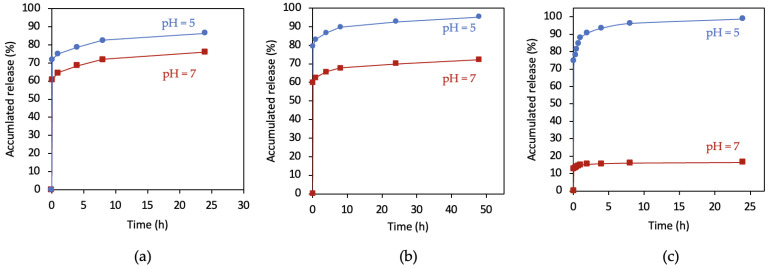
Release profiles of ZIF-8-encapsulated CIP in neutral and acid media: (**a**) CIP/ZIF (0.007 mg/mg); (**b**) CIP/ZIF (0.4 mg/mg); and (**c**) CIP/ZIF (2 mg/mg).

The UV spectra were obtained for each material released at different time points, performing the corresponding calculations and creating the release graphs shown in Figure 9.

Figure 9 depicts the early release of virtually all of the antibiotic in both media, followed by a gradual and stable release over the next hours.

The most significant difference between neutral and acid media was in the total release of antibiotics from ZIF-8-encapsulated CIP, which was higher in acid versus neutral pH at all three drug loads studied. This is because the ZIF-8 compound degrades more completely and more quickly at slightly an acid pH than at a physiological-neutral pH. This difference is greater with a higher drug load and is much more marked for the highest load (Figure 9c), with the release of only 20% of the content at a neutral pH versus almost 100% in an acid pH over the same time period. This reflects the collapse of the structure of ZIF-8 at a slightly acid pH, which has been described by other authors [22]. A very early stabilization was observed, which was slightly slower at the acid pH. At normal pH 7, the CIP %release for CIP/ZIF loadings of 0.007, 0.4, 0.6, and 2 mg/mg are 75%, 70%, 30%, and 20%, respectively. This may indicate that at CIP/ZIF loadings greater than about 0.5 mg/mg, CIP accumulates on the surface of ZIF and form a protective layer for the ZIF structure by bonding at the surface. This bonding is broken in the acid (pH 5) environment which helps the collapse of the ZIF structure, and the CIP %release jumps from 20–30% to 90–100%.

Specifically, the amounts of CIP released by CIP/ZIF (0.007 mg/mg) were highly similar between pH 7 and pH 5 (0.11 mg vs. 0.12 mg), while a greater difference (5.4 mg vs. 7.1 mg) was observed for the material with a medium load (0.4 mg/mg), and the greatest difference was with a high load (6.5 mg vs. 39.5 mg). This suggests that there is a “critical” drug dose from which ZIF-8 truly acts as a drug release regulator in an acidified medium.

In short, encapsulation in ZIF-8 permits the targeted release of CIP at an acid pH, releasing a larger amount of the drug in this medium. An initial and early explosive release is observed, followed by a sustained release over time.

### 3.3. CIP Encapsulated in Magnetic ZIF-8

#### 3.3.1. ZIF-8 Magnetization

**Figure 10:**
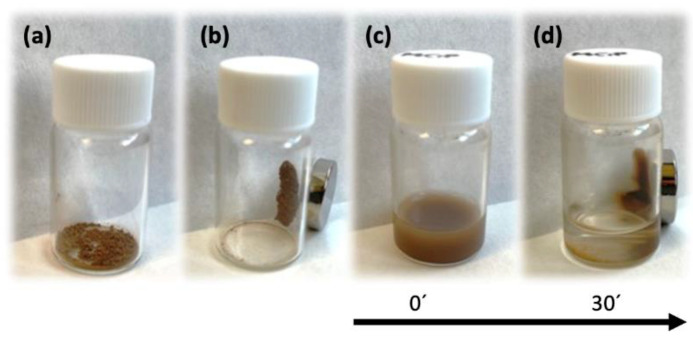
Magnetization test of dry magnetic CIP/ZIF (0.6 mg/mg) with (**a**) and without (**b**) magnet; the same material in water with (**c**) and without (**d**) magnet.

Figure 10 depicts the images of ZIF-8 with encapsulated CIP next to a magnet. The magnetic effect is immediate on the powdered material (Figure 10a,b) and is slower but equally effective in aqueous solution (Figure 10c,d), achieving complete separation of material from solution in <30 min. These magnetic properties allow targeting of the biological environment to be treated and markedly increase the traceability of the drug by means of soft magnetic fields.

#### 3.3.2. Release Profiles of CIP Encapsulated in Magnetic ZIF-8

**Figure 11:**
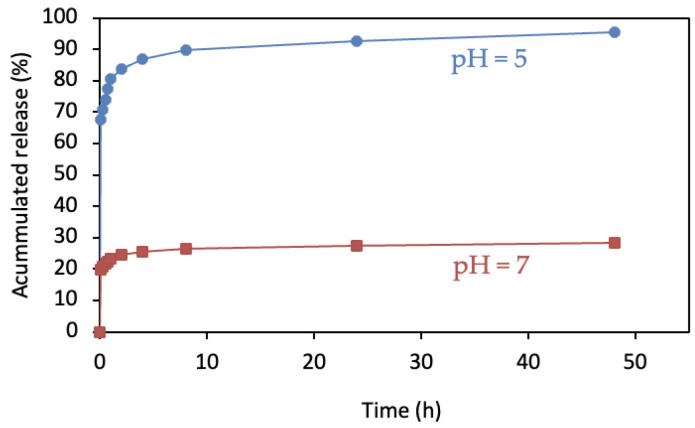
Release profiles of magnetic CIP/ZIF (0.6 mg/mg) in neutral and acid media.

A similar release profile was observed between magnetic and non-magnetic ZIF-8: an extremely abrupt release during the first few minutes of the reaction with subsequent slowing and stabilization over time. A comparison of profiles between acid and neutral pH (Figure 11) again showed a markedly higher percent of material released in the former (3.1 mg vs. 11.0 mg, respectively). ZIF-8 can, therefore, be magnetized without modifying its release kinetics at neutral or acid pH values. Hence, magnetic MOF can be used to direct the drug to affected areas for release at the appropriate site, offering a rapid, localized, and targeted route of action.

### 3.4. Release Kinetics

Table 1 shows the mathematical parameters of the models used to interpret the release kinetics together with the correlation coefficient R^2^. It is possible to appreciate that both the zero-order model, as well as the first one, presented a poor adjustment of the experimental data. On the other hand, the Higuchi and Korsmeyer–Peppas models, whose theoretical foundations are linked to mass transfer processes, presented a better interpretation. In the case of the Higuchi model, it can be seen in general that as the CIP dose increases, the values of the kinetic constant KH also increase. In addition, when correlating the quotient of the kinetic constants at both pH values with the dose of CIP in the material (KHpH5/KHpH7 vs. CIP/ZIF), a trend of exponential increase is observed; this can be associated with the gradient of the driving force of concentration and diffusion processes analyzed with Fick’s law. On the other hand, except for the CIP/ZIF material (0.007 mg/mg), the kinetic constant at pH 5 presented higher values than those reported at pH 7; this may be associated with the disintegration of the ZIF-8 structure observed at pH = 5.

The Korsmeyer–Peppas model, also known as Power law, was also used, with mathematical consideration for an abrupt increase in the initial release of CIP. This model was the one that presented a better adjustment of the experimental data with an average correlation coefficient R^2^ equal to 0.97. For this model, it has been reported that for values of n < 0.5, the release mechanism can be associated with Fick-type mass transport mechanisms; such is the case of our study, since for all cases the value of the exponent varied between 0.186 and 0.198. On the other hand, parameter b, associated with the burst-type release effect, presented quasi-constant values of 0.27 and 0.37 for pH values of 7 and 5, respectively. This increase in the burst-like release rate is clearly seen in Figure 9 and Figure 11 and may be associated with the collapse of the ZIF-8 structure at an acid pH, as previously discussed. Finally, when analyzing the kinetic constant of the KPL model, average values around 0.473 and 0.392 can be seen for pH values of 7 and 5, respectively. This decrease in the constant is reflected in a more developed curvature in the release profiles and can be associated with a decrease in the driving force, as the concentration gradient decreases more significantly in the experiments carried out at pH 5.

The kinetic parameters of the magnetized ZIF-8 sample presented the same behavior as the unmagnetized materials, so the inclusion of the magnetic component does not seem to alter the release capacities of the pharmaceutical compound, indicating that there is no interaction between CIP and iron nanoparticles

### 3.5. Antimicrobial Activity Test

Finally, results were obtained for the antimicrobial activity of each synthesized compound (Table 2) by measuring the inhibition halo for *Staphylococcus aureus* and *Escherichia coli*, measured in mm.

These data indicate that ZIF-8 per se has antimicrobial activity, especially against Gram-positive bacteria, which is virtually equal to that of CIP alone. In addition, the antibacterial activity of CIP encapsulated in ZIF-8 is slightly higher than in the adsorbed CIP, being even greater for the highest encapsulated CIP loads than that of the blank (32 and 42 mm CIP/ZIF-8 in 1000 mg vs. 31 and 27 mm of CIP alone). We also underline the virtually null activity of iron nanoparticles alone and their higher activity when incorporated in CIP/ZIF-8, even exceeding that of CIP alone.

## 4. Conclusions

The use of MOFs for drug transport is attracting increased research interest. Their unique crystalline structures provide porous compounds for housing such molecules as drugs or therapeutic agents. ZIF-8 was selected for study because of its textural properties, stability, and low toxicity. ZIF-8 proved suitable for housing drugs of large molecular size, using CIP as a model. Three materials were prepared with CIP encapsulated at low (0.007 mg/mg), medium (0.4 mg/mg), and high (2 mg/mg) doses as well as a magnetic CIP/ZIF composite with intermediate dose of 0.6 mg/mg. Physicochemical characterization showed high material crystallinity and thermal stability up to 300 °C. In addition, the antimicrobial capacity of the CIP/ZIF system against *E. coli* and *S. aureus* exceeded that of pure CIP, exerting a synergic effect.

ZIF-8 degrades when in contact with slightly acidic medium (around pH = 5). The Fe_3_O_4_ nanoparticles inhibition of ZIF structures’ aggregation and the smaller ZIF structures will increase the effective surface area to increase the drug loading than would be released at normal pH 7 but has rapid release at the (pH 5) acidified diseases cells. It is, therefore, of special interest for the delivery and controlled release of therapeutic agents that course with cell acidification. Future research is warranted on the encapsulation and localized release of anticancer drugs (e.g., methotrexate and paclitaxel) because carcinogenic cells acidify their surroundings, favoring the targeted release of these agents using ZIF-8 as vehicle.

Initial drug release was greater (by up to 80%) and more abrupt at an acid versus a neutral pH. This would be a suitable profile for diseases that course with cell acidification and require a first shock dose followed by a lower maintenance dose but not for those requiring the release of a given dose over time. However, the system could be optimized for the latter, avoiding the explosive initial release through functionalization with hydrophobic groups or groups that promote stronger bonds with the drug.

This study also demonstrates the possibility of directing delivery of the material to its place of action by using magnetic fields. A possible disadvantage of these compounds would be aggregation, hampering their parenteral administration. However, this study shows the possibility of optimizing the material by using different solvents and controlling the reaction time, with the option of scaling it to nanometric size, hence facilitating its future dosage.

In conclusion, the use of MOFs as drug-transporting materials represents a major advance in the treatment of diseases that course with varied physiological changes by increasing selectivity, reducing possible adverse effects, and possibly decreasing the required dose.

## Figures and Tables

**Table 1:** Kinetic constants and characteristic parameters obtained after applying the kinetic models.

Material		Zero Order		First Order		Higuchi Model		Korsmeyer–Peppas Model			
		K_0_ mg L^−1^ h^−1^	R^2^	K_1_ h^−1^	R^2^	K_H_ mg h^−0.5^	R^2^	K	n	b	R^2^
CIP/ZIF (0.007 mg/mg)	pH 7	0.1556	0.11	0.0180	0.77	0.0044	0.96	0.4641	0.1943	0.2645	0.99
	pH 5	0.2142	0.22	0.0152	0.79	0.0043	0.96	0.3865	0.1869	0.3616	0.99
CIP/ZIF (0.40 mg/mg)	pH 7	4.1755	0.19	0.0073	0.73	0.1320	0.92	0.4439	0.1903	0.2587	0.99
	pH 5	5.4427	0.18	0.0070	0.72	0.1671	0.92	0.3850	0.1862	0.3600	0.99
CIP/ZIF (2 mg/mg)	pH 7	0.0081	0.12	0.0163	0.49	0.2701	0.75	0.5267	0.1979	0.2849	0.91
	pH 5	52.8240	0.14	0.0198	0.50	1.9422	0.76	0.4132	0.1882	0.3848	0.92
Magnetic CIP/ZIF (0.6 mg/mg)	pH 7	2.3750	0.19	0.0135	0.56	0.1386	0.80	0.4581	0.1933	0.2624	0.94
	pH 5	7.6077	0.17	0.0123	0.51	0.4329	0.76	0.3850	0.1862	0.3600	0.92

**Table 2:** Antimicrobial activity of the materials studied by inhibition halo measurement.

Bacterial Inhibition Diameter (mm)		
	*E. coli*	*S. aureus*
CIP Blank	31	27
ZIF-8	12	24
CIP/ZIF (0.007 mg/mg)	21	15
CIP/ZIF (0.4 mg/mg)	24	38
CIP/ZIF (2 mg/mg)	32	42
Nanoparticles (NP) Fe	9	0
ZIF-8 with NP Fe	26	14
Magnetic CIP/ZIF (0.6 mg/mg)	37	30

## Data Availability

Not applicable.

## Supplementary Materials

The following supporting information can be downloaded at: https://www.mdpi.com/article/10.3390/pharmaceutics14112546/s1, Figure S1: EDX analysis of (a) ZIF-8 (5min, synthesis in water); (b) ZIF-8 (24 h, synthesis in water); (c) ZIF-8 (5min, synthesis in acetone); (d) ZIF-8 (24 h, synthesis in acetone); (e) CIP/ZIF (0,007 mg/mg); (f) CIP/ZIF (2 mg/mg); (g) magnetic ZIF-8 and (h) magnetic CIP/ZIF (0.6 mg/mg); Figure S2: Simulated XRD pattern of pure magnetite (Fe_3_O_4_) obtained from the Open Crystallography Database (COD), COD number: 9007644.
