# Supplementary material for: ZIF-8 and Its Magnetic Functionalization as Vehicle for the Transport and Release of Ciprofloxacin
Source: Pharmaceutics. 2022 Nov 21;14(11):2546. doi: 10.3390/pharmaceutics14112546 (PMC9693427; doi:10.3390/pharmaceutics14112546)
Supplement: Supplementary file 1 [file pharmaceutics-14-02546-s001.zip › pharmaceutics-2022258-supplementary.pdf]

pharmaceutics

Supporting Information

ZIF-8 and Its Magnetic Functionalization as Vehicle for the Transport and Release of Ciprofloxacin

Ventura Castillo Ramos [1,*], Cinthia Berenice García Reyes [1,2], Guillermo Mangas García [1], María Inmaculada Sampedro Quesada [3], Fernando José Martínez-Checa Barrero [3,*], Jacob Josafat Salazar Rábago [2] andManuel Sánchez Polo [1]

[1] Department of Inorganic Chemistry, Faculty of Science, University of Granada, 18071 Granada, Spain
[2] Faculty of Chemical Sciences, Nuevo León Autonomous University, San Nicolás de los Garza 66455, Mexico
[3] Department of Microbiology, Faculty of Science, University of Granada, 18071 Granada, Spain
* Correspondence: vcastillo@ugr.es (V.C.R); fmcheca@ugr.es (F.J.M-C.B.)

S1. Energy Dispersive X-ray Analysis (EDX) Results

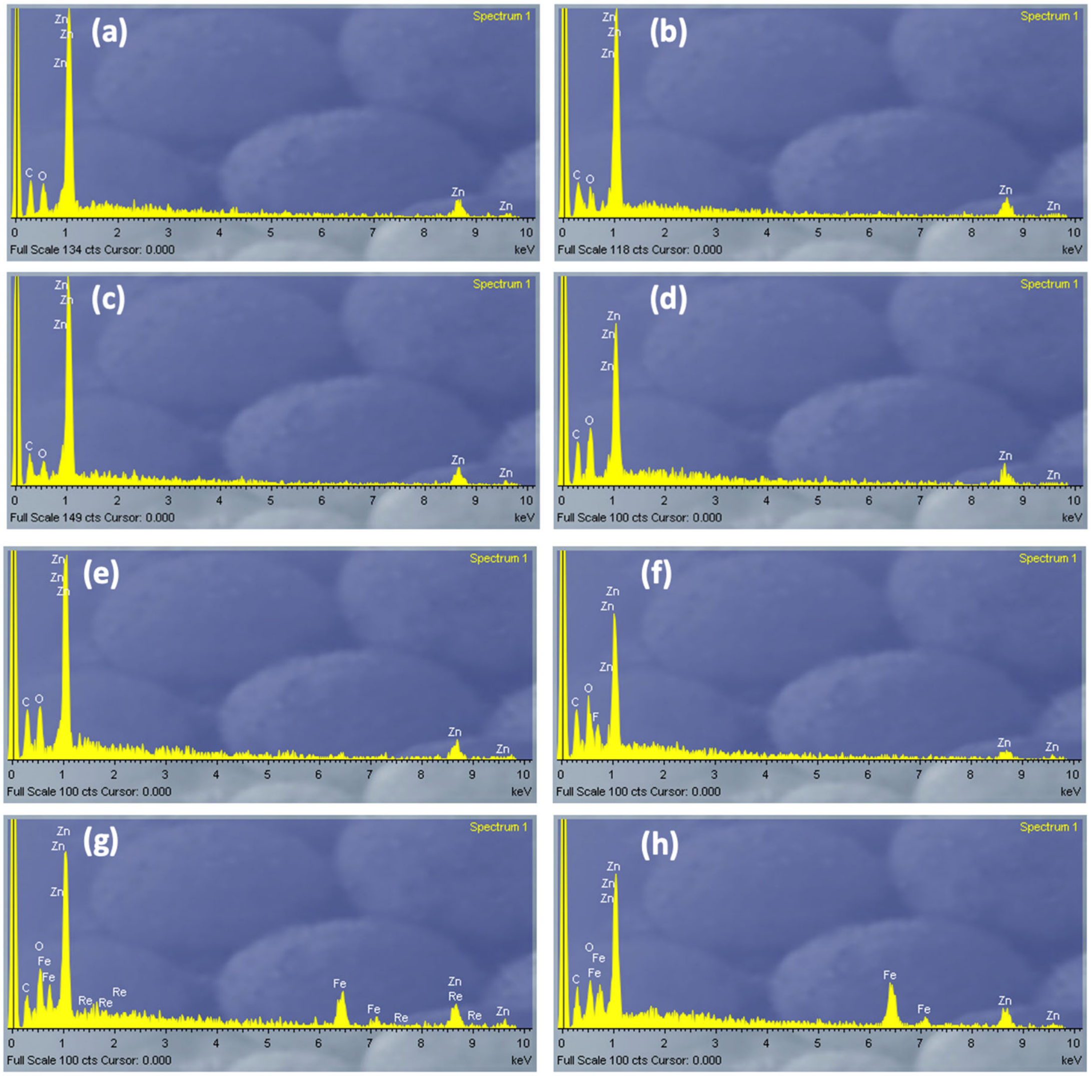

Figure S1. EDX analysis of (a) ZIF-8 (5min, synthesis in water); (b) ZIF-8 (24 h, synthesis in water); (c) ZIF-8 (5min, synthesis in acetone); (d) ZIF-8 (24 h, synthesis in acetone); (e) CIP/ZIF (0,007 mg/mg); (f) CIP/ZIF (2 mg/mg); (g) magnetic ZIF-8 and (h) magnetic CIP/ZIF (0.6 mg/mg).

S2. Kinetic Models

S2.1. Zero-Order Kinetic Model

The zero-order model, in terms of concentration, is described, in its linear form, by Equation (S1)[1]:

$$C_t = C_0 + K_0 t \quad \text{(S1)}$$

Where C_t represents the amount of drug released during time t, C_0 is the initial drug concentration (usually 0), and K_0 is the zero-order constant. In this model, drug release is only a function of time and the process occurs at a constant rate independent of drug concentration.

By plotting the experimentally measured concentrations (C_t) over time and linear regression, it was possible to obtain the value of the zero-order constant (slope of the line).

S2.2. First Order Kinetic Model

The first-order kinetic model, in its general form, states that the change in concentration with respect to time is dependent only on concentration [1]:

$$\frac{dC}{dt} = -KC \quad \text{(S2)}$$

where C is the drug concentration and K is the first-order release constant. Differentiating and linearizing the equation, the following expression can be obtained:

$$logQ_1 = logQ_0 + \frac{k_1 t}{2.303} \quad \text{(S3)}$$

Where Q_1 is the amount of drug released at time t, Q_0 is the initial amount of drug dissolved, and k_1 is the first-order constant. Plotting $logQ_1$ vs. time, it was possible to determine the first-order kinetic constant, from the slope, which corresponds to $K_1/2.303$.

S2.3. Higuchi´s Kinetic Model

Higuchi's model, in its linear form, states that the amount of drug released is proportional to the square root of time [1]:

$$Q = K_H \sqrt{t} \quad \text{(S4)}$$

where Q is the amount of drug released and K_H is the Higuchi release constant. The assumptions that must be followed for the use of the Higuchi model are:

- The drug-carrying matrix contains an initial concentration of drug much greater than its solubility.
- Diffusion is unidirectional, because edge effects are negligible.
- The thickness of the drug dispenser is much greater than the size of the drug molecules.
- The swelling or dissolution of the drug-carrying matrix is negligible.
- The diffusivity of the drug is constant.

Representing the experimental values of release with the square root of time, it was possible to determine the values of the constant K_H by linear regression.

S2.4. Korsmeyer-Peppas´ Kinetic Model

It is a semi-empirical model that establishes an exponential relationship between drug release and time [1]:

$$f_1 = \frac{M_i}{M_\infty} = Kt^n \quad \text{(S5)}$$

Where f_1 is the amount of drug released, M_∞ is the amount of drug at equilibrium (normally close to the amount of drug contained in the carrier material at the beginning of the release process), M_i is the amount of drug released over time t, K is the rate constant and n is the release exponent (related to the release mechanism). Furthermore, when the drug release process is characterized by an abrupt release at the beginning of the release, the following equation was proposed:

$$\frac{M_i}{M_\infty} = Kt^n + b \quad \text{(S6)}$$

Where b is the "burst effect".

Through non-linear regression using the Excel Solver tool, minimizing the squared error between the experimental and modeled values, it was possible to determine the factors b and n, as well as the kinetic constant of the model.

S3. Simulated XRD Patter of Magnetite (Fe_3O_4)

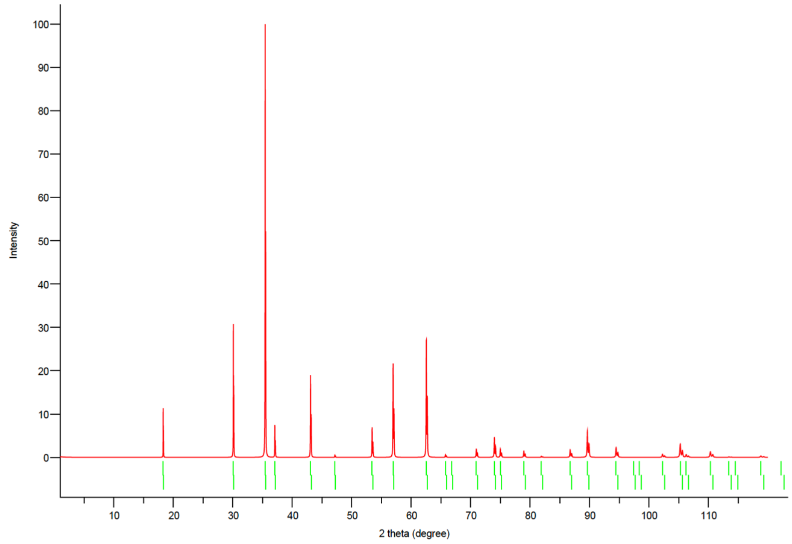

Figure S2. Simulated XRD pattern of pure magnetite (Fe_3O_4) obtained from the Open Crystallography Database (COD), COD number: 9007644.

References

[1] Marcos Luciano Bruschi, "Strategies to Modify the Drug Release from Pharmaceutical Systems," Strategies to Modify the Drug Release from Pharmaceutical Systems, pp. 87–194, 2015, Accessed: Jun. 24, 2022. [Online]. Available: http://www.sciencedirect.com/science/article/pii/B9780081000922000060
